# Plasticity Is Key to Success of *Drosophila suzukii* (Diptera: Drosophilidae) Invasion

**DOI:** 10.1093/jisesa/ieaa034

**Published:** 2020-05-17

**Authors:** Catherine M Little, Thomas W Chapman, N Kirk Hillier

**Affiliations:** 1 Department of Biology, Acadia University, Wolfville, NS, Canada; 2 Department of Biology, Memorial University of Newfoundland and Labrador, St. John’s, NL, Canada

**Keywords:** invasive species, alternative host, plant phylogeny, spotted-wing Drosophila

## Abstract

After its initial discovery in California in 2008, *Drosophila suzukii* Matsumura has become one of the most important invasive agricultural pest insects across climate zones in much of Asia, Europe, North America, and South America. Populations of *D. suzukii* have demonstrated notable behavioral and physiological plasticity, adapting to diverse environmental and climatic conditions, interspecific competition, novel food sources, and potential predators. This adaptability and plasticity have enabled rapid range expansion and diversified niche use by *D. suzukii*, making it a species particularly suited to changing habitats and conditions. This article reviews factors and evidence that influence plasticity in *D. suzukii* and promotes this species’ invasiveness.

Plasticity is a driving force behind the spread of numerous invasive species. Plasticity refers to the degree to which traits of individuals or populations can rapidly adapt to new or changing environmental conditions (Sgrò et al. 2016). Adaptations can be in the form of phenotypic, behavioral, developmental, or physiological traits.

Resistance to or tolerance of stressful conditions and a short generation time increase the risk of unintentional transportation and facilitate introduction (Gippet et al. 2019). Newly introduced species often suffer from genetic bottlenecks, which can be offset by plasticity. Once introduced into a new environment, such phenotypic or behavioral plasticity can promote an exotic species to becoming established in its new environment by exploiting vulnerable niches in these habitats (Sakai et al. 2001, Engel et al. 2011, Garnas et al. 2016). Introduced species with limited plasticity in key traits would be compromised in their ability to adapt to new environments, including novel biotic and abiotic factors (Chown et al. 2007, Engel et al. 2011). Plasticity can further promote the continued spread of newly established species beyond its point of introduction (Sakai et al. 2001).

Physiological or behavioral plasticity can result from differences in environmental conditions (e.g., temperature, humidity, photoperiod), available diet, or pressure from predators or competitors (Hamby et al. 2016, Wallingford et al. 2016, Guédot et al. 2018). This plasticity can take many forms, including the ability to exploit novel food resources, as in the Oriental fruit moth, *Grapholita molesta* (Busck) (Lepidoptera: Tortricidae), which is able to use many apple varieties as oviposition substrates, or the ability to outcompete local species, as in the Asian ladybird, *Harmonia axyridis* (Pallas) (Coleoptera: Coccinellidae), which develop more quickly than the North American native ladybird, *Adalia bipunctata* L. (Coleoptera: Coccinellidae) (Beukeboom 2018).

*Drosophila suzukii* Matsumura was first described in Japan (Matsumura 1931) but is believed to have originated in mainland Asia. Shortly thereafter, it was identified as the source of damage in fruit crops in Japan (Kanzawa 1935, 1939). By 1980, *D. suzukii* had been confirmed in Hawai’i (Kaneshiro 1983, O’Grady et al. 2002, Leblanc et al. 2009). *Drosophila suzukii* was first reported in North America in California in 2008 and has since spread across the continental United States, north into Canada, and south into Mexico (Hauser 2011, Walsh et al. 2011). Since 2008, *D. suzukii* has also invaded most of Europe and South America (Calabria et al. 2012, Cini et al. 2014, Deprá et al. 2014, Benito et al. 2016, Andreazza et al. 2017). *Drosophila suzukii* is now well established throughout most subtropical, temperate, and boreal regions (Andreazza et al. 2017, Dos Santos et al. 2017, Little et al. 2017, Manduric 2017). *Drosophila suzukii* is now considered one of the most important agricultural pest species throughout most of its invasive range (Benito et al. 2016, Gutierrez et al. 2016).

*Drosophila suzukii* possess a number of traits that predispose it to dispersal and unintentional introductions to new environments (Gippet et al. 2019). For example, the species is closely associated with small fruit species that are routinely transported internationally. All life stages of *D. suzukii* are small and inconspicuous. Additionally, eggs and larvae are usually located within the fruits and are not visible during external examination. Thus, *D. suzukii* is less likely to be detected during transportation of these fruits or upon arrival in new geographic regions (Gippet et al. 2019). Since 2008, *D. suzukii* has been highly successful at moving from unintentional introductions to become invasive species due in large part to notable plasticity in development, adult phenotype, and behavior (Jakobs et al. 2015, 2017; Hamby et al. 2016; Shearer et al. 2016; Fraimout et al. 2018; Stockton et al. 2018).

## Phenotypic Plasticity

### Morphological Plasticity

At its most basic level, phenotypic plasticity refers to differences in individual morphological traits in response to environmental conditions, diet, or other factors (Moczek 2010). Colder temperatures during slow larval development resulting in adult *D. suzukii* with larger wings, which allows cold-reared flies to accelerate and fly faster, but not for greater duration, than flies reared in warmer temperatures (Shearer et al. 2016, Fraimout et al. 2018). In contrast, warmer temperatures during development result in smaller wings and smaller wing spots in male flies, which fly less quickly (Fraimout et al. 2018, Varón-González et al. 2020). Thus, cold-reared *D. suzukii* could be able to disperse faster and further during flights of the same duration compared with flies reared under more moderate temperatures, potentially further increasing the risk of increased range expansion in temperate and boreal regions. In mark–recapture trials in Trentino, Italy, winter morph *D. suzukii* were recovered more than 9,000 m from a point source (Tait et al. 2018). Larval diet can induce differences in adult morphology, including wing size and shape (Pajač Živković et al. 2018). Furthermore, differences in wing morphology as populations of *D. suzukii* experience seasonal and regional differences in fruit availability can influence flight ability and potential dispersal distances. Diet also affects mating behavior of adult *D. suzukii*, which when reared on suboptimal diet are both less selective of and less successful at attracting potential mates (Young et al. 2017). Therefore, such environmental drivers which subsequently alter wing morphology of *D. suzukii* may result in positive, negative, or neutral impacts on fitness.

At its most extreme, morphological plasticity is revealed as polyphenism, where distinct phenotypes are expressed in response to different conditions (Moczek 2010). In subtropical regions, where environmental conditions are suitable year-round, *D. suzukii* are active year-round (Harris et al. 2014, Andreazza et al. 2017, Dos Santos et al. 2017). In temperate regions, mated adult females overwinter as reproductively quiescent winter morphs rather than experiencing true reproductive diapause (Dalton et al. 2011, Shearer et al. 2016, Toxopeus et al. 2016, Guédot et al. 2018). Acclimation to cold temperatures and shorter photoperiods promotes cold tolerance in both adult and pupal *D. suzukii*, improving survival of the induced winter morph adults (Wallingford et al. 2016, Stockton et al. 2018). Winter morph flies are more cold-tolerant and usually larger than summer morphs (Stephens et al. 2015, Shearer et al. 2016, Wallingford and Loeb 2016). Given favorable diet, postoverwintering female winter morph *D. suzukii* are longer lived and have greater fecundity than summer morph flies; however, when diet is suboptimal, fecundity and longevity of summer morph flies are less restricted by low temperatures (Rendon et al. 2018, 2019).

## Developmental Plasticity

### Temperature and Desiccation Tolerance

A species that demonstrates an ability to adapt to a range of temperature and humidity conditions can more readily become established in novel habitats. Individuals or populations can acquire increased tolerance to temperature extremes through hardening (short-term exposure), acclimation (long-term exposure in a laboratory setting), or acclimatization (long-term exposure in a natural setting; Sinclair et al. 2015).

Generation time of *D. suzukii* is approximately 12–15 d; however, development time is dependent on temperature and larval diet (Stockton et al. 2019a). Larvae emerge from eggs within 12–72 h of oviposition and progress through three instars. Larvae develop most quickly at 26–28°C (Kinjo et al. 2014, Tochen et al. 2014, Asplen et al. 2015). Temperatures below this range or fluctuating temperatures can slow larval development to as much as 64 d (Jakobs et al. 2017).

Exposure to fluctuating temperatures during development induces greater cold tolerance in adult flies (Stephens et al. 2015, Stockton et al. 2018). This cold tolerance is due in part to increased accumulation of cryoprotectant compounds (Enriquez et al. 2018). Despite this, freezing will kill larvae and adults, and both third-instar larvae and adults are chill susceptible (Jakobs et al. 2015, 2017; Enriquez and Colinet 2017; Stockton et al. 2018). Temperatures between 22.6 and 28.2°C are optimal for *D. suzukii* development; however, larval development and adult emergence can occur within a wider range of 8.1–30.9°C (Tochen et al. 2014, Ryan et al. 2016). Adult activity, including oviposition behavior, is limited below 10°C (Wallingford et al. 2016, Zerulla et al. 2017, Leach et al. 2019). Complete development is most reliable at constant temperatures of 20–26°C (Kinjo et al. 2014, Tochen et al. 2014, Asplen et al. 2015).

Pupae are more tolerant of extreme heat than adult *D. suzukii*, provided heat stress is not compounded with low humidity (Enriquez and Colinet 2017). Fifty percent of pupae can survive temperatures as high as 37°C for up to 4 h (Enriquez and Colinet 2017). Temperatures during development also affects adult morphology, particularly wing size and shape, which in turn affects flight ability (Fraimout et al. 2018). Heat stress reduces adult life span, fecundity, and reproductive activity, as evidenced by a lack of oviposition behavior at temperatures of 33°C and above (Enriquez and Colinet 2017, Evans et al. 2018, Kirk Green et al. 2019). Male *D. suzukii* are more susceptible to effects of heat stress than are female flies (Kirk Green et al. 2019). Egg viability, pupal development, and adult eclosion were also compromised above 28°C (Evans et al. 2018, Kirk Green et al. 2019).

Oviposition and successful larval development can occur at temperatures as low as 11.1°C (Tonina et al. 2016). Although larvae and pupae are not able to survive prolonged temperatures below 5°C, adult winter morph *D. suzukii* survival has been reported to survive continuous 6-wk exposure at temperatures as low as 1°C (Ryan et al. 2016; Stockton et al. 2018, 2019a). Survival of adult *D. suzukii* at colder temperatures could be possible when temperatures fluctuate, allowing for repair of cold damage during warmer periods in a mechanism similar to that observed in cold-acclimated *Alphitobius diaperinus* Panzer (Coleoptera: Tenebrionidae) (Renault et al. 2004). Adult *D. suzukii* can survive 1-h exposure to temperatures as low as −7.5°C (Jakobs et al. 2015, Stockton et al. 2018). Acclimation to cold temperatures improves both survival during short-term exposure and duration of survivable exposure (Jakobs et al. 2015). Acclimation to cold temperatures induces upregulation of up to 1,583 genes, including genes for ion transport, cellular signaling, and carbohydrate metabolism, while also inducing downregulation of an additional 1,325 genes, including genes for oogenesis (Shearer et al. 2016, Enriquez and Colinet 2019). Thus, exposure to cold temperatures can result in epigenetic changes in physiology that promote metabolic homeostasis and enable increased tolerance to more extreme environmental conditions (Enriquez et al. 2018). Five days after cold shock exposure, fecundity of female *D. suzukii* returns to pre-exposure levels (Plantamp et al. 2016).

Gradual acclimation to cold temperatures and low humidity may interact to further facilitate cold tolerance (Guédot et al. 2018, Stockton et al. 2018). Acclimation to cold temperatures allows adult *D. suzukii* to survive temperatures below 0°C for longer periods, withstand chill coma symptoms at lower temperatures, and recover from cold exposure more quickly (Jakobs et al. 2015). Acclimation to cold temperatures at both developmental and adult stages infers chill protection in adult *D. suzukii* through greater homeostatic stability due to accumulated cryoprotectant amino acids and carbohydrates (Enriquez et al. 2018). Female winter morph *D. suzukii* that survive initial exposure to low humidity conditions are able to withstand continued dry conditions for longer periods than summer morph flies (Fanning et al. 2019).

Low humidity levels limit survival of all life stages of *D. suzukii* (Tochen et al. 2014, 2016a; Gutierrez et al. 2016). Low relative humidity decreases both fecundity and longevity in *D. suzukii* (Tochen et al. 2014, 2016a; Guédot et al. 2018; Fanning et al. 2019). Attempts to limit ambient humidity in crop areas through irrigation practices or crop system management can be of limited efficacy, because low relative humidity does not limit *D. suzukii* flight distance or duration (Wong et al. 2018, Rendon and Walton 2019). Additionally, humidity levels in and below organic mulches, such as sawdust or woodchip, can be higher than above the mulch surface, potentially providing suitable conditions for pupal development (Rendon and Walton 2019). However, effects of mulch on *D. suzukii* adult emergence during field trials are inconclusive (Rendon et al. 2020). Natural refuges, including accumulations of leaf litter, as well as microclimates in and around built-structures permit increased survival during extreme winter weather conditions (Zerulla et al. 2015, Gutierrez et al. 2016, Wallingford et al. 2018, Stockton et al. 2019a). Overwintered female *D. suzukii* have been found bearing mature eggs as early in spring as at 7 degree-days and begin ovipositioning in the first available fruits of spring at only 87 degree-days (Grassi et al. 2018, Panel et al. 2018). Therefore, adult *D. suzukii* can survive and reproduce in less-favorable environments through behavioral plasticity, by making short-distance movements in and out of nearby more favorable microclimates (Klick et al. 2016, Tochen et al. 2016a).

It is apparent that *D. suzukii* can adapt physiologically and behaviorally to tolerate a wide range of temperature and humidity conditions, particularly if those conditions are localized or transient. As the effects of climate change become more pronounced, fluctuations in both temperature and humidity will be more prevalent over a wider geographic area, promoting further opportunities for *D. suzukii* to find favorable habitats. *Drosophila suzukii* could also use marginally suitable habitats, provided more favorable microclimate areas are accessible within their flight range.

## Behavioral Plasticity

### Plasticity in Circadian Activity

Locomotor activity of *D. suzukii* is mediated by light conditions, ambient temperatures, and relative humidity, with flies most active at dawn/dusk during summer conditions and at the warmest portion of the day during winter conditions (Hamby et al. 2013, Evans et al. 2017, Hansen et al. 2019, Shaw et al. 2019). Social interactions within groups of flies increases synchronicity of activity among individuals and reinforces locomotor activity patterns, particularly crepuscular activity and movements among localized microclimates, which would further promote increased grouping of flies (Hansen et al. 2019, Shaw et al. 2019). Localized populations exposed to different microclimate conditions could experience shifts in gene allele frequencies and differences in behavior that could ultimately lead to microevolutionary changes among populations. Upregulation of detoxification transcription factors also fluctuates in response to an endogenous circadian clock, which results in daily periods of increased and decreased pesticide susceptibility (Hamby et al. 2013). Preliminary research suggests that *D. suzukii* is not at peak insecticide susceptibility during peak periods of activity in crop areas (Hamby et al. 2013).

Activity levels of female *D. suzukii* vary with their mating status. Virgin flies of both sexes are quiescent in mid-afternoon to reduce exposure to sun and heat (Ferguson et al. 2015). However, mated female flies remain active throughout this period, tolerating both heat and lower humidity (Ferguson et al. 2015). Gravid female *D. suzukii* oviposit greater numbers of eggs when temperatures are between 25 and 28°C and will shift timing of oviposition behavior based on daily temperature fluctuations (Kinjo et al. 2014, Evans et al. 2017). Fly activity on fruit and in flight near fruit plants is flexible depending on temperature and humidity levels, but *D. suzukii* behavior is largely unaffected by irrigation and insecticide application procedures (Van Timmeren et al. 2017).

### Olfactory Plasticity

Although *D. suzukii* is more sensitive than *Drosophila melanogaster* Meigan (Diptera: Drosophilidae) to volatiles produced by ripening fruit, there is evidence to suggest *D. suzukii* also uses differences in leaf tissue volatiles produced during fruit development as a supplemental means to locate potential feeding and oviposition sites (Keesey et al. 2015, Bolton et al. 2019). In laboratory studies, *D. suzukii* demonstrates unique antennal response profiles to fruit and yeast associated odorants that enable identification of ripening fruits and sugar receptors that enable identification of floral nectars (Scheidler et al. 2015, Hickner et al. 2016). Preference for volatile odors differ depending on the sex and physiological state (mated or unmated females) of adult *D. suzukii* and on environmental states (temperature and ambient temperatures; Wong et al. 2018, Clymans et al. 2019). Gravid female *D. suzukii* select oviposition sites using a combination of chemosensory cues, including olfactory, tactile, and potential gustatory signals (Karageorgi et al. 2017). Environmental odors can reduce *D. suzukii* attraction to otherwise desirable olfactory cues, which can further hamper the effort to identify effective olfactory attractant lures for monitoring purposes (Cloonan et al. 2019). Male and unmated female *D. suzukii* prefer volatiles associated with fermentation and indicative of high-protein food sources; however, mated female flies prefer fruit odors indicative of substrates more suitable for oviposition sites (Karageorgi et al. 2017, Wong et al. 2018, Clymans et al. 2019). However, as with other phytophagous insects, previous experience can induce host acceptance or alter host preference hierarchies (Jaenike 1990, Anderson and Anton 2014). Although *D. suzukii* larvae reared on blackberry (*Rubus* L. subgenus *rubus* Watson [Rosales: Rosaceae]) had no oviposition preference as adults between blackberry and American pokeweed (*Phytolacca americana* L. [Caryophyllales: Phytolaccaceae]), flies reared on American pokeweed preferred to oviposit on blackberry (Diepenbrock et al. 2016, Stockton et al. 2019a).

Differences between summer and winter morph flies also extends to physiological and behavioral responses to odorant stimuli. Summer morph *D. suzukii* are more responsive to ecologically relevant volatile odorants, including both potential attractant and deterrent compounds (Kirkpatrick et al. 2018). This responsiveness in summer morph individuals is reflective of greater activity for host-finding for adult feeding and also of host-finding for oviposition sites. Winter morph *D. suzukii* undergo a reproductive diapause and search out food sources during periods when ambient temperatures make volatiles are less prevalent. Winter morph flies prefer shelter sites containing food sources; either for winter feeding or as a protein source for egg development as they emerge from reproductive quiescence (Wallingford et al. 2018).

### Dietary Plasticity

*Drosophila suzukii* are polyphagous and highly adaptable. Beukeboom (2018) identified the propensity of a species to identify and use alternative hosts as oviposit sites as a critical determinant of its invasiveness. Availability (and/or apparency) of suitable host plants and breadth of diet usually have an inverse relationship (see Jaenike 1990). Plant species differ in terms of chemistry, physical characteristics, and phenology. To date, infestation by *D. suzukii* has been confirmed in 198 plant species representing 73 genera in 39 angiosperm families and two genera in one gymnosperm family (Supp Table 1 and 2 [online only]). In addition, in 41 instances, host fruits had been identified to genus rather than species level (Supp Table 1 [online only]). Preferences among fruit species have been documented; however, it is evident that host choice is to some extent opportunistic (Lee et al. 2011, Burrack et al. 2013, Poyet et al. 2015, Little et al. 2017, Stockton et al. 2019a). Seasonal availability due to plant phenology is a key factor in risk of damage from *D. suzukii* (Wiman et al. 2014, Haviland et al. 2016, Kenis et al. 2016). Plants that produce fruit in spring or early summer, such as gooseberries, and early-season strawberries and cherries, are less vulnerable than are plants that fruit in late summer or autumn in temperate regions when *D. suzukii* populations are larger (Wiman et al. 2014, Kenis et al. 2016). Recent evidence suggests that *D. suzukii* prefer wounded fruits for adult feeding and prefer healthy undamaged fruits for oviposition sites, but when availability of healthy fruits is limited, *D. suzukii* will accept damaged fruits as acceptable egg-laying site substitutes (Kienzle et al. 2020). Selecting for less susceptible varieties of a fruit species can help growers mitigate crop damage (Sward et al. 2016). Early-ripening varieties of blueberries and raspberries could be harvested before *D. suzukii* populations reach threshold densities. Soft fruits and berries with firmer flesh or tougher skins could withstand oviposition efforts. Fruit varieties that do not change color until late stages of ripeness or that complete the ripening process after harvest would be less conspicuous and potentially limit attraction cues. For example, early harvesting of wild blueberries can limit fruit infestation, but incurs additional costs due to lost immature fruit (Drummond et al. 2019). Given the extreme plasticity in host-use by *D. suzukii*, growers need to assess fruit breeding and cultivation practices to limit risk of infestation. Removal of windfall and damaged fruits could reduce access by *D. suzukii* to potential reproductive sites post-harvest (Bal et al. 2017, Kienzle et al. 2020). In semitropical regions, warm temperatures early in the calendar year permit more rapid increases in *D. suzukii* populations and putting early-season fruit crops at greater risk (Wiman et al. 2014).

Characters associated fruit species have been correlated with suitability for feeding and oviposition behavior of *D. suzukii.* Sweetness (Brix), skin toughness, and acidity (pH) have been investigated most commonly (Lee et al. 2011, 2016; Burrack et al. 2013; Little et al. 2017). Other factors have been explored, including fruit size, fruit shape, fruit texture (peach indumenta or strawberry accessions), fruit ripeness stage, fruit odors, fruit color, damage to fruit, fruit phenology, location of fruit relative to rest of plant (height, outer vs inner part of plant), leaf coverage (obscured vs exposed), hanging versus fallen fruit, and competition (previous oviposition by conspecifics or heterospecifics; Stewart et al. 2014, Gong et al. 2016, Haviland et al. 2016, Sward et al. 2016, Rice et al. 2017, Cha et al. 2018, Little et al. 2018, Thistlewood et al. 2018). *Drosophila suzukii* are able to use a wide variety of host fruits by selecting for characteristics which signal health and ripeness of host fruits and a lack of potential competitors, pathogens, or predators. Other plant characters, such as leaf odors, leaf color, and overall health of plant, have received less attention, but are important supplemental cues used to locate host fruits of healthy plants (Keesey et al. 2015, Little et al. 2018, Bolton et al. 2019). Foraging behavior and responses to food cues can be modified by the composition of the fly’s own gut microbiota (Wong et al. 2017).

Larval development time varies depending on larval diet. Larvae feeing on fruits such as cherry, blueberry, or raspberry, develop more quickly than those that feed on standard diet media (Jaramillo et al. 2015, Hamby et al. 2016). Larvae reared on raspberry and blackberry are better able to withstand competitive pressures than larvae reared on other fruits (Olazcuaga et al. 2019). Naturally occurring yeasts and fungi associated with fruit provide developing *D. suzukii* larvae with critical nutrients, including protein, vitamins, and minerals (Hamby and Becher 2016, Bellutti et al. 2018, Lewis et al. 2018). As with other *Drosophila* species, yeast quality affects development, fecundity, and lifespan of *D. suzukii* (Hamby and Becher 2016, Bellutti et al. 2018, Grangeteau et al. 2018). However, unlike many other *Drosophila* species, *D. suzukii* larvae develop most successfully on foods containing lower ratios of protein to carbohydrates, developing more quickly into larger adults with greater potential fecundity (Jaramillo et al. 2015; Silva-Soares et al. 2017; Rendon et al. 2018, 2019; Young et al. 2018).

In temperate regions, fruit can be of limited availability or quality as a food source for adult flies and as an oviposition site. In laboratory studies, adult *D. suzukii* have been documented feeding on floral nectar, tree sap, and honeydew when other food sources were unavailable (Kanzawa 1939, Lee et al. 2015, Tochen et al. 2016b, Wong et al. 2018, Stockton et al. 2019b). In the absence of suitable fruits, female *D. suzukii* will also oviposit and larvae can successfully develop on less ideal materials, including mushrooms and chicken manure (Stockton et al. 2019b). Thus, the adaptability of *D. suzukii* to novel dietary choices, for both the adult and larval stages, contributes to its invasion success and interact with other aspects of its plasticity.

### Plasticity in Community Interactions

Interspecific and intraspecific competition can induce changes in oviposition behavior of female adult *D. suzukii* and in behavior of larvae. Chemical cues or signs of previous oviposition by heterospecifics, such as *D. melanogaster*, can deter female *D. suzukii* from ovipositing in the same fruit (Shaw et al. 2018, Kidera and Takahashi 2020). This may be due to repellent olfactory cues such as trace amounts of the male *D. melanogaster* sex pheromone, *cis*-vaccenyl acetate, transferred to fruit during oviposition. In direct interspecific competition situations, *D. melanogaster* larvae have greater survival than *D. suzukii* larvae (Gao et al. 2018). This is due in part to higher tolerance to ethanol produced through decay and fermentation of fruit damaged by larval feeding and that higher levels of ethanol are produced in fruit containing *D. melanogaster* larvae than fruit containing *D. suzukii* larvae (Sampson et al. 2016, Gao et al. 2018). Although *D. suzukii* females prefer to oviposit in ripe fruit, they are able to shift ecological niches and use ripening fruit to avoid competitive pressures and reduce potential ethanol exposure to their larvae.

In contrast, female *D. suzukii* were not deterred from ovipositing in fruit containing eggs from other *D. suzukii* females (Shaw et al. 2018). In response to intraspecific competition, *D. suzukii* larvae are less likely to remain within their original host fruits throughout their development. In the absence of competition, larvae remain on or in host fruits throughout pupation. However, as the number of intraspecific competitors within a fruit host increases, larvae move greater distances away from the fruit to pupate (Bezerra Da Silva et al. 2019).

In the presence of parasitoid populations, *D. suzukii* select fruit for oviposition that contain atropine compounds to infer prophylactic protection to the next generation (Poyet et al. 2017). This is similar behavior to *D. melanogaster*’s use of ethanol laden oviposition sites following detection of parasites and parasitoids (Milan et al. 2012, Kacsoh et al. 2013).

## Summary

Success of an introduced species in a novel environment depends primarily on its ability to adapt and explore its surroundings (Fordyce 2006). Species introduced into locations with changeable environmental conditions, such as temperate regions, are especially reliant on plastic morphological, behavioral, and physiological characteristics for survival (Fordyce 2006). Novel environments expose species to different potential food sources, competitors, and predators.

The relatively small size and inconspicuous nature of *D. suzukii* makes it easily overlooked or misidentified in field settings. Without close inspection, it is easily mistaken for native Drosophilids in each of its invasive regions. Although no voucher specimens exist in mainland American entomological collections prior 2008, it is highly likely that *D. suzukii* was introduced to North America or Europe long before its first recognized detection in California, but it was not present in sufficient numbers to cause significant crop damage or invite notice (Hauser 2011). Effects of escalating climate change and extinction of native insect species could be contributing factors in promoting ecological niche availability beneficial to *D. suzukii* invasion (Ward and Masters 2007, Rhodes 2019).

*Drosophila suzukii* has demonstrated a notable ability to adapt behaviorally, physiologically, and morphologically to new environments. Although each of these responses is of themselves modest in scope, the nature of its adaptive responses is arguably more important to its success as an invasive species than is the extent of any one variable response (Chown et al. 2007). *Drosophila suzukii* has shown itself to be highly opportunistic and it has been able to adapt to a wide range of host plant fruit for rearing its larvae and for adult feeding. As a result of its willingness to explore and test novel fruit species, it has become extremely polyphagous. In addition, *D. suzukii* has adapted to a wide range of temperature and humidity conditions, through its behavior and through physiological, developmental, and morphological plasticity. As *D. suzukii* has expanded its range into temperate regions, its ability to adapt to changing environmental conditions and then reverse those adaptations as conditions change through successive seasons as been vital to its success. Plasticity within multiple aspects of behavior, physiology, and morphology has allowed *D. suzukii* to move from a localized introduced species to an established invasive species over a global range.

## Supplementary Material

ieaa034_suppl_Supplementary_Tables
