# Supplementary material for: Plasticity Is Key to Success of Drosophila suzukii (Diptera: Drosophilidae) Invasion
Source: J Insect Sci. 2020 May 17;20(3):5. doi: 10.1093/jisesa/ieaa034 (PMC7230767; doi:10.1093/jisesa/ieaa034)
Supplement: ieaa034_suppl_Supplementary_Tables [file ieaa034_suppl_supplementary_tables.docx]

**Supplementary information**

**Plasticity is key to success of *Drosophila suzukii* (Diptera: Drosophilidae) invasion.**

Catherine M. Little^1,2^, Thomas W. Chapman^2^, and N. Kirk Hillier^1^

**Affiliations**

^1^ Department of Biology, Acadia University, Wolfville NS Canada B4P2R6

^2^ Department of Biology, Memorial University of Newfoundland and Labrador, St. John’s NL Canada A1C5S7

**Supplementary Table S1.** Confirmed host plants for *D. suzukii*.

| **Family** | **Genus** | **Species** | **Location** | **Reference** | |
| --- | --- | --- | --- | --- | --- |
| Actinidiaceae | Actinidia | *Actinidia arguta* | OR USA | Lee et al. 2015 | |
|  |  |  | NS Canada | Little et al. 2019 | |
|  |  | *Actinidia chinensis* | Italy | Kenis et al. 2016 | |
| Adoxaceae | Sambucus | *Sambucus canadensis* | NS Canada | Little et al. 2019 | |
|  |  |  | MN USA | Sward (Thesis) 2017 | |
|  |  |  | MI USA | Leach et al. 2019 | |
|  |  | *Sambucus ebulus* | Italy | Kenis et al. 2016 | |
|  |  |  | France | Poyet et al. 2015 | |
|  |  | *Sambucus nigra* | Italy | Grassi et al. 2011 | |
|  |  |  | OR USA | Lee et al. 2015 | |
|  |  |  | France | Poyet et al. 2015 | |
|  |  |  | Slovenia | Seljak et al. 2015 | |
|  |  |  | Italy | Grassi et al. 2011 | |
|  |  |  | Spain | Arnó et al. 2016 | |
|  |  |  | Italy | Kenis et al. 2016 | |
|  |  |  | Netherlands | Kenis et al. 2016 | |
|  |  |  | Switzerland | Kenis et al. 2016 | |
|  |  |  | Italy | Tonina et al. 2016 | |
|  |  |  | BC Canada | Thistlewood et al. 2019 | |
|  |  | *Sambucus nigra* cerulea | BC Canada | Thistlewood et al. 2019 | |
|  |  | *Sambucus racemosa* | Italy | Kenis et al. 2016 | |
|  |  |  | Netherlands | Kenis et al. 2016 | |
|  |  |  | Switzerland | Kenis et al. 2016 | |
|  |  |  | Italy | Tonina et al. 2016 | |
|  |  | *Sambucus racemosa* melanocarpa | BC Canada | Thistlewood et al. 2019 | |
|  |  | *Sambucus* spp | AR USA | Herrera (Thesis) 2017 | |
|  |  |  | Sweden | Manduric 2017 | |
|  |  |  | MI USA | Lee et al. 2015 | |
| **Family** | **Genus** | **Species** | **Location** | **Reference** | |
|  | Viburnum | *Viburnum cassinoides* | NS Canada | Little et al. 2019 | |
|  |  | *Viburnum dilatatum* | Japan | Mitsui et al. 2010 | |
|  |  | *Viburnum lantana* | Italy | Kenis et al. 2016 | |
|  |  | *Viburnum opulus* | Slovenia | Seljak et al. 2015 | |
|  |  | *Viburnum rhytidophyllum* | Netherlands | Kenis et al. 2016 | |
| Aquifoliaceae | Ilex | *Ilex mucronata* | ME USA | Ballman and Drummond 2017 | |
| Araceae | Arum | *Arum italicum* | Netherlands | Kenis et al. 2016 | |
|  |  | *Arum maculatum* | France | Poyet et al. 2015 | |
| Araliaceae | Hedera | *Hedera helix* | Italy | Grassi et al. 2018 | |
|  |  |  | Netherlands | Panel et al. 2018 | |
| Arecaceae | Butia | *Butia eriospatha* | Brazil | Andreazza et al. 2015 | |
| Asparagaceae | Polygonatum | *Polygonatum multiflorum* | Netherlands | Kenis et al. 2016 | |
| Basellaceae | Basella | *Basella alba* | NC USA | Diepenbrock and McPhie 2018 | |
| Berberidaceae | Berberis | *Berberis aquifolium* (=*Mahonia aquifolium*) | OR USA | Lee et al. 2015 | |
|  |  |  | Netherlands | Kenis et al. 2016 | |
|  |  |  | BC Canada | Thistlewood et al. 2019 | |
|  |  |  | France | Poyet et al. 2015 | |
|  |  | *Berberis thunbergii* (=*Mahonia thunbergii*) | MI USA | Leach et al. 2019 | |
|  |  | *Berberis hortensis* (=*Mahonia x media*) | France | Poyet et al. 2015 | |
|  |  | *Berberis* spp. (=*Mahonia* spp.) | Italy | Kenis et al. 2016 | |
|  |  |  | BC Canada | Thistlewood et al. 2019 | |
| Buxaceae | Sarcococca | *Sarcococca confusa* | OR USA | Lee et al. 2015 | |
| Caprifoliaceae | Lonicera | *Lonicera alpigena* | Italy | Kenis et al. 2016 | |
|  |  |  | Italy | Tonina et al. 2016 | |
|  |  | *Lonicera caerulea* | Italy | Kenis et al. 2016 | |
|  |  |  | OR USA | Lee et al. 2015 | |
|  |  |  | NS Canada | Little et al. 2020 | |
|  |  |  | Italy | Tonina et al. 2016 | |
| **Family** | **Genus** | **Species** | **Location** | **Reference** | |
|  |  | *Lonicera caprifolium* | Netherlands | Kenis et al. 2016 | |
|  |  | *Lonicera ferdinandii* | Netherlands | Kenis et al. 2016 | |
|  |  | *Lonicera japonica* | ME USA | Ballman and Drummond 2017 | |
|  |  | *Lonicera maackii* | AR USA | Herrera (Thesis) 2017 | |
|  |  |  | AR USA | Knipp (Thesis) 2018 | |
|  |  |  | MI USA | Leach et al. 2019 | |
|  |  | *Lonicera morrowii* | MA USA | Elsensohn and Loeb 2018 | |
|  |  |  | MN USA | Sward (Thesis) 2017 | |
|  |  |  | MI USA | Leach et al. 2019 | |
|  |  | *Lonicera nigra* | Italy | Kenis et al. 2016 | |
|  |  |  | Italy | Tonina et al. 2016 | |
|  |  | *Lonicera nitida* | Netherlands | Kenis et al. 2016 | |
|  |  |  | France | Poyet et al. 2015 | |
|  |  | *Lonicera sempervirens* | AR USA | Knipp (Thesis) 2018 | |
|  |  | *Lonicera* spp*.* | Italy | Grassi et al. 2011 | |
|  |  |  | AR USA | Herrera (Thesis) 2017 | |
|  |  |  | Switzerland | Kenis et al. 2016 | |
|  |  |  | MI USA | Lee et al. 2015 | |
|  |  | *Lonicera tatarica* | MN USA | Sward (Thesis) 2017 | |
|  |  |  | MI USA | Leach et al. 2019 | |
|  |  |  | BC Canada | Thistlewood et al. 2019 | |
|  |  | *Lonicera xylosteum* | France | Poyet et al. 2015 | |
|  |  |  | Italy | Tonina et al. 2016 | |
|  |  |  | Italy | Kenis et al. 2016 | |
|  | Symphoricarpos | *Symphoricarpos albus* | Netherlands | Kenis et al. 2016 | |
|  |  |  | OR USA | Lee et al. 2015 | |
|  |  |  | France | Poyet et al. 2015 | |
|  |  |  | BC Canada | Thistlewood et al. 2019 | |
| Cornaceae | Alangium | *Alangium platanifolium* | Japan | Mitsui et al. 2010 | |
|  | Cornus | *Cornus alba* | Netherlands | Kenis et al. 2016 | |
| **Family** | **Genus** | **Species** | **Location** | **Reference** | |
|  |  | *Cornus amomum* | MI USA | Lee et al. 2015 | |
|  |  |  | MA USA | Elsensohn and Loeb 2018 | |
|  |  |  | MI USA | Leach et al. 2019 | |
|  |  | *Cornus canadensis* | ME USA | Ballman and Drummond 2017 | |
|  |  | *Cornus controversa* | Japan | Mitsui et al. 2010 | |
|  |  | *Cornus foemina* | MI USA | Lee et al. 2015 | |
|  |  | *Cornus kousa* | Netherlands | Kenis et al. 2016 | |
|  |  |  | OR USA | Lee et al. 2015 | |
|  |  |  | Japan | Mitsui et al. 2010 | |
|  |  | *Cornus mas* | Italy | Kenis et al. 2016 | |
|  |  |  | Netherlands | Kenis et al. 2016 | |
|  |  | *Cornus racemosa* | MN USA | Sward (Thesis) 2017 | |
|  |  | *Cornus sanguinea* | Italy | Kenis et al. 2016 | |
|  |  |  | Netherlands | Kenis et al. 2016 | |
|  |  | *Cornus sericea* | OR USA | Lee et al. 2015 | |
|  |  |  | France | Poyet et al. 2015 | |
|  |  | *Cornus* spp. | ME USA | Ballman and Drummond 2017 | |
| Cucurbitaceae | Bryonia | *Bryonia cretica* | Spain | Arnó et al. 2016 | |
|  | Cucurbita | *Cucurbita pepo* | MI USA | Bal et al. 2017 | |
| Dioscoreaceae | Dioscorea | *Dioscorea communis (=Tamus communis)* | Italy | Kenis et al. 2016 | |
|  |  |  | Switzerland | Kenis et al. 2016 | |
| Ebenaceae | Diospyros | *Diospyros kaki* | Japan | Kanzawa 1935 | |
|  |  |  | Japan | Kanzawa 1939 | |
|  |  |  | Japan | Mitsui et al. 2010 | |
|  |  |  | Slovenia | Seljak et al. 2015 | |
| Elaeagnaceae | Elaeagnus | *Elaeagnus multiflora* | Japan | Kanzawa 1939 | |
|  |  |  | Japan | Sasaki and Sato 1995 | |
|  |  | *Elaeagnus x ebbingei* | Netherlands | Panel et al. 2018 | |
|  |  |  | France | Poyet et al. 2015 | |
| **Family** | **Genus** | **Species** | **Location** | **Reference** | |
|  |  | *Elaeagnus* spp. | AR USA | Herrera (Thesis) 2017 | |
|  |  | *Elaeagnus umbellata* | MI USA | Lee et al. 2015 | |
|  |  |  | MI USA | Leach et al. 2019 | |
|  | Hippophae | *Hippophae rhamnoides* | Switzerland | Kenis et al. 2016 | |
|  |  |  | NS Canada | Little et al. 2017 | |
|  |  |  | France | Poyet et al. 2015 | |
| Ericaceae | Arbutus | *Arbutus unedo* | Spain | Arnó et al. 2012 | |
|  |  |  | OR USA | Lee et al. 2015 | |
|  |  |  | Italy | Kenis et al. 2016 | |
|  | Empetrum | *Empetrum nigrum* | NS Canada | Little et al. 2020 | |
|  | Vaccinium | *Vaccinium angustifolium* | ME USA | Ballman and Drummond 2017 | |
|  |  |  | NS Canada | Little et al. 2020 | |
|  |  | *Vaccinium ashei* | Uruguay | González et al. 2015 | |
|  |  | *Vaccinium corymbosum* | Italy | Grassi et al. 2011 | |
|  |  |  | Japan | Kinjo et al. 2013 | |
|  |  |  | Slovenia | Seljak et al. 2015 | |
|  |  |  | NS Canada | Little et al. 2017 | |
|  |  |  | MN USA | Sward (Thesis) 2017 | |
|  |  |  | NS Canada | Little et al. 2018 | |
|  |  |  | NS Canada | Little et al. 2019 | |
|  |  |  | NS Canada | Little et al. 2020 | |
|  |  | *Vaccinium macrocarpon* | WI USA | Steffan et al. 2013 | |
|  |  |  | NS Canada | Little et al. 2017 | |
|  |  | *Vaccinium myrtilloides* | Netherlands | Kenis et al. 2016 | |
|  |  | *Vaccinium myrtillus* | Slovenia | Seljak et al. 2015 | |
|  |  |  | Italy | Kenis et al. 2016 | |
|  |  |  | Switzerland | Kenis et al. 2016 | |
|  |  | *Vaccinium oldhamii* | Netherlands | Kenis et al. 2016 | |
|  |  | *Vaccinium ovatum* | OR USA | Lee et al. 2015 | |
|  |  | *Vaccinium praestans* | Netherlands | Kenis et al. 2016 | |
| **Family** | **Genus** | **Species** | **Location** | **Reference** | |
|  |  | *Vaccinium* spp. | Japan | Mitsui et al. 2010 | |
|  |  |  | CA USA | Haviland et al. 2016 | |
|  |  |  | Sweden | Manduric 2017 | |
|  |  |  | Netherlands | Panel et al. 2018 | |
|  |  | *Vaccinium uliginosum* | France | Poyet et al. 2015 | |
|  |  | *Vaccinium virgatum* | Japan | Kinjo et al. 2013 | |
|  |  | *Vaccinium vitis-idaea* | OR USA | Lee et al. 2015 | |
|  |  |  | Netherlands | Kenis et al. 2016 | |
|  |  |  | NS Canada | Little et al. 2017 | |
|  | Gaultheria | *Gaultheria adenothrix* | Japan | Mitsui et al. 2010 | |
|  |  | *Gaultheria shallon* | OR USA | Lee et al. 2015 | |
|  |  | *Gaultheria x wisleyensis* | Netherlands | Kenis et al. 2016 | |
| Garryaceae | Aucuba | *Aucuba japonica* | Japan | Mitsui et al. 2010 | |
|  |  |  | Netherlands | Panel et al. 2018 | |
|  |  |  | France | Poyet et al. 2015 | |
| Grossulariaceae | Ribes | *Ribes aureum* | BC Canada | Thistlewood et al. 2019 | |
|  |  | *Ribes hudsonianum var. petiolare* | BC Canada | Thistlewood et al. 2019 | |
|  |  | *Ribes nigrum* | MI USA | Leach et al. 2019 | |
|  |  | *Ribes rubrum* | Netherlands | Kenis et al. 2016 | |
|  |  |  | Sweden | Manduric 2017 | |
|  |  |  | France | Poyet et al. 2015 | |
|  |  | *Ribes sanguineum* | France | Poyet et al. 2015 | |
|  |  | *Ribes uva-crispa* | OR USA | Lee et al. 2015 | |
| Iridaceae | Iris | *Iris* spp. | France | Poyet et al. 2015 | |
| Lamiaceae | Callicarpa | *Callicarpa americana* | AR USA | Knipp (Thesis) 2018 | |
| Lauraceae | Lindera | *Lindera benzoin* | MI USA | Lee et al. 2015 | |
| Melanthiaceae | Paris | *Paris quadrifolia* | Switzerland | Kenis et al. 2016 | |
| Menispermaceae | Cocculus | *Cocculus carolinus* | AR USA | Herrera (Thesis) 2017 | |
| Moraceae | Ficus | *Ficus carica* | Slovenia | Seljak et al. 2015 | |
| **Family** | **Genus** | **Species** | **Location** | **Reference** | |
|  |  |  | Italy | Kenis et al. 2016 | |
|  |  |  | Switzerland | Kenis et al. 2016 | |
|  |  |  | CA USA | Yu et al. 2013 | |
|  |  | *Ficus* spp. | Italy | Grassi et al. 2011 | |
|  | Morus | *Morus alba* | Japan | Kanzawa 1939 | |
|  |  | *Morus alba x rubra* | CA USA | Yu et al. 2013 | |
|  |  | *Morus australis* (=*bombycis*) | Japan | Mitsui et al. 2010 | |
|  |  | *Morus nigra* | OR USA | Lee et al. 2015 | |
|  |  |  | Slovenia | Seljak et al. 2015 | |
|  |  | *Morus rubra* | AR USA | Knipp (Thesis) 2018 | |
|  |  |  | FL USA | Plant Inspection Advisory 2010 | |
|  |  | *Morus* spp. | Japan | Kanzawa 1935 | |
|  |  |  | Japan | Sasaki and Sato 1995 | |
|  |  |  | France | Poyet et al. 2015 | |
|  |  |  | AR USA | Herrera (Thesis) 2017 | |
|  |  |  | Argentina | Lavagnino et al. 2018 | |
| Myricaceae | Myrica | *Myrica rubra* (=*Morella rubra*) | Japan | Yukinari 1988 | |
| Myrtaceae | Acca | *Acca sellowiana* | Brazil | Souza et al. 2017 | |
|  | Psidium | *Psidium cattleyanum* | Brazil | Andreazza et al. 2015 | |
|  |  |  | Brazil | Andreazza et al. 2017 | |
|  |  | *Psidium guajava* | Mexico | Lasa et al. 2017 | |
|  |  |  | Brazil | Andreazza et al. 2015 | |
|  | Eugenia | *Eugenia involucrata* | Brazil | Andreazza et al. 2017 | |
|  |  | *Eugenia uniflora* | FL USA | Plant Inspection Advisory 2010 | |
|  |  |  | Brazil | Andreazza et al. 2015 | |
|  |  |  | Brazil | Andreazza et al. 2017 | |
| Oleaceae | Ligustrum | *Ligustrum lucidum* | Slovenia | Seljak et al. 2015 | |
|  |  | *Ligustrum vulgare* | Slovenia | Seljak et al. 2015 | |
| Onagraceae | Fuchsia | *Fuchsia spp.* | France | Poyet et al. 2015 | |
| Phytolaccaceae | Phytolacca | *Phytolacca americana* | Japan | Sasaki and Sato 1995 | |
| **Family** | **Genus** | **Species** | **Location** | **Reference** | |
|  |  |  | France | Poyet et al. 2015 | |
|  |  |  | MI USA | Lee et al. 2015 | |
|  |  |  | Italy | Kenis et al. 2016 | |
|  |  |  | Switzerland | Kenis et al. 2016 | |
|  |  |  | AR USA | Herrera (Thesis) 2017 | |
|  |  |  | AR USA | Knipp (Thesis) 2018 | |
|  |  |  | MI USA | Leach et al. 2019 | |
|  |  |  | MA USA | Elsensohn and Loeb 2018 | |
|  |  | *Phytolacca esculenta* | Netherlands | Kenis et al. 2016 | |
| Punicaceae | Punica | *Punica granatum* | Slovenia | Seljak et al. 2015 | |
| Rhamnaceae | Frangula | *Frangula alnus* | Italy | Grassi et al. 2011 | |
|  |  |  | Italy | Kenis et al. 2016 | |
|  |  |  | Netherlands | Kenis et al. 2016 | |
|  |  |  | Switzerland | Kenis et al. 2016 | |
|  |  |  | France | Poyet et al. 2015 | |
|  |  | *Frangula caroliniana* | AR USA | Herrera (Thesis) 2017 | |
|  |  | *Frangula purshiana* | OR USA | Lee et al. 2015 | |
|  | Rhamnus | *Rhamnus caroliniana* | AR USA | Knipp (Thesis) 2018 | |
|  |  | *Rhamnus cathartica* | MA USA | Elsensohn and Loeb 2018 | |
|  |  |  | Netherlands | Kenis et al. 2016 | |
|  |  |  | MN USA | Sward (Thesis) 2017 | |
|  |  | *Rhamnus fallax* | Slovenia | Seljak et al. 2015 | |
| Rosaceae | Amelanchier | *Amelanchier alnifolia* | NS Canada | Little et al. 2020 | |
|  |  | *Amelanchier lamarckii* | Netherlands | Kenis et al. 2016 | |
|  |  | *Amelanchier ovalis* | Italy | Kenis et al. 2016 | |
|  | Aronia | *Aronia melanocarpa* | KS USA | Hietala-Henschell et al. 2017 | |
|  | Cotoneaster | *Cotoneaster apiculatus* | MI USA | Leach et al. 2019 | |
|  |  | *Cotoneaster* *bullatus* | France | Poyet et al. 2015 | |
|  |  | *Cotoneaster franchetii* | Netherlands | Kenis et al. 2016 | |
|  |  | *Cotoneaster lacteus* | Italy | Kenis et al. 2016 | |
| **Family** | **Genus** | **Species** | **Location** | **Reference** | |
|  |  |  | OR USA | Lee et al. 2015 | |
|  |  | *Cotoneaster rehderi* | Netherlands | Kenis et al. 2016 | |
|  |  | *Cotoneaster watereri* | France | Poyet et al. 2015 | |
|  | Duchesnea | *Duchesnea indica* | Italy | Kenis et al. 2016 | |
|  |  |  | Netherlands | Kenis et al. 2016 | |
|  |  |  | Switzerland | Kenis et al. 2016 | |
|  |  |  | France | Poyet et al. 2015 | |
|  |  |  | OR USA | Lee et al. 2015 | |
|  | Eriobotrya | *Eriobotrya japonica* | Japan | Kanzawa 1939 | |
|  |  |  | FL USA | Plant Inspection Advisory 2010 | |
|  |  |  | Italy | Kenis et al. 2016 | |
|  |  |  | Brazil | Andreazza et al. 2017 | |
|  | Fragaria x ananassa hybrid | *Fragaria × ananassa* | Italy | Grassi et al. 2011 | |
|  |  |  | CA USA | Goodhue et al. 2012 | |
|  |  |  | Slovenia | Seljak et al. 2015 | |
|  |  |  | NS Canada | Little et al. 2017 | |
|  |  |  | NS Canada | Little et al. 2020 | |
|  |  |  | Brazil | Andreazza et al. 2017 | |
|  | Fragaria | *Fragaria vesca* | Italy | Kenis et al. 2016 | |
|  |  |  | Netherlands | Kenis et al. 2016 | |
|  |  |  | Switzerland | Kenis et al. 2016 | |
|  |  |  | France | Poyet et al. 2015 | |
|  |  | *Fragaria* spp. | Sweden | Manduric 2017 | |
|  | Malus | *Malus baccata* | Netherlands | Kenis et al. 2016 | |
|  |  | *Malus domestica* | NS Canada | Little et al. 2019 | |
|  |  | *Malus pumila* | Japan | Kanzawa 1939 | |
|  |  |  | NS Canada | Little et al. 2019 | |
|  |  | *Malus sylvestris* | France | Poyet et al. 2015 | |
| **Family** | **Genus** | **Species** | **Location** | **Reference** | |
|  |  | *Malus* spp. | MI USA | Bal et al. 2017 | |
|  |  |  | MI USA | Leach et al. 2019 | |
|  | Photinia | *Photinia beauverdiana* | Netherlands | Kenis et al. 2016 | |
|  |  | *Photinia prunifolia* | Netherlands | Kenis et al. 2016 | |
|  |  | *Photinia villosa* | Netherlands | Kenis et al. 2016 | |
|  | Prunus | *Prunus armeniaca* | Japan | Kanzawa 1935 | |
|  |  |  | Japan | Kanzawa 1939 | |
|  |  |  | Slovenia | Seljak et al. 2015 | |
|  |  |  | Italy | Kenis et al. 2016 | |
|  |  | *Prunus avium* | Japan | Kanzawa 1939 |  |
|  |  |  | Italy | Grassi et al. 2011 |  |
|  |  |  | France | Poyet et al. 2015 |  |
|  |  |  | OR USA | Lee et al. 2015 |  |
|  |  |  | Slovenia | Seljak et al. 2015 |  |
|  |  |  | CA USA | Haviland et al. 2016 |  |
|  |  |  | Netherlands | Kenis et al. 2016 |  |
|  |  |  | Switzerland | Kenis et al. 2016 | |
|  |  |  | Italy | Grassi et al. 2018 | |
|  |  |  | NS Canada | Little et al. 2017 | |
|  |  |  | NS Canada | Little et al. 2020 | |
|  |  | *Prunus buergeriana* | Japan | Sasaki and Sato 1995 | |
|  |  | *Prunus cerasifera* | Italy | Kenis et al. 2016 | |
|  |  | *Prunus cerasus* | Japan | Kanzawa 1939 | |
|  |  |  | Slovenia | Seljak et al. 2015 | |
|  |  |  | Italy | Kenis et al. 2016 | |
|  |  | *Prunus domestica* | Slovenia | Seljak et al. 2015 | |
|  |  |  | Switzerland | Kenis et al. 2016 | |
|  |  | *Prunus donarium* | Japan | Kanzawa 1939 | |
|  |  |  | Japan | Mitsui et al. 2006 | |
|  |  | *Prunus japonica* | Japan | Kanzawa 1935 | |
| **Family** | **Genus** | **Species** | **Location** | **Reference** | |
|  |  |  | Japan | Kanzawa 1939 | |
|  |  | *Prunus laurocerasus* | Sweden | Manduric 2017 | |
|  |  |  | Italy | Kenis et al. 2016 | |
|  |  |  | Netherlands | Kenis et al. 2016 | |
|  |  |  | Switzerland | Kenis et al. 2016 | |
|  |  |  | OR USA | Lee et al. 2015 | |
|  |  | *Prunus lusitanica* | Italy | Kenis et al. 2016 | |
|  |  |  | OR USA | Lee et al. 2015 | |
|  |  |  | France | Poyet et al. 2015 | |
|  |  | *Prunus mahaleb* | Japan | Kanzawa 1935 | |
|  |  |  | Japan | Kanzawa 1939 | |
|  |  |  | France | Poyet et al. 2015 | |
|  |  |  | Spain | Arnó et al. 2016 | |
|  |  |  | Italy | Kenis et al. 2016 | |
|  |  |  | BC Canada | Thistlewood et al. 2019 | |
|  |  | *Prunus nipponica* | Japan | Mitsui et al. 2010 | |
|  |  | *Prunus padus* | Netherlands | Kenis et al. 2016 | |
|  |  |  | Switzerland | Kenis et al. 2016 | |
|  |  | *Prunus persica* | Japan | Kanzawa 1935 | |
|  |  |  | Japan | Kanzawa 1939 | |
|  |  |  | Japan | Sasaki and Sato 1995 | |
|  |  |  | CA USA | Stewart et al. 2014 | |
|  |  |  | Slovenia | Seljak et al. 2015 | |
|  |  |  | Brazil | Andreazza et al. 2017 | |
|  |  |  | MI USA | Bal et al. 2017 | |
|  |  | *Prunus persica* nucipersica | Slovenia | Seljak et al. 2015 | |
|  |  | *Prunus pensylvanica* | NS Canada | Little et al. 2020 | |
|  |  | *Prunus salicina (=triflora)* | Japan | Kanzawa 1935 | |
|  |  |  | Japan | Kanzawa 1939 | |
|  |  | *Prunus sargentii* | Japan | Kanzawa 1935 | |
| **Family** | **Genus** | **Species** | **Location** | **Reference** | |
|  |  | *Prunus serotina* | PA USA | Turcotte et al. 2018 | |
|  |  |  | AR USA | Herrera (Thesis) 2017 | |
|  |  |  | Netherlands | Kenis et al. 2016 | |
|  |  |  | France | Poyet et al. 2014 | |
|  |  |  | France | Poyet et al. 2015 | |
|  |  | *Prunus spinosa* | France | Poyet et al. 2015 | |
|  |  |  | Italy | Kenis et al. 2016 | |
|  |  |  | Netherlands | Kenis et al. 2016 | |
|  |  |  | Sweden | Manduric 2017 | |
|  |  | *Prunus* spp. | Japan | Mitsui et al. 2010 | |
|  |  |  | Italy | Grassi et al. 2011 | |
|  |  |  | MI USA | Bal et al. 2017 | |
|  |  |  | ME USA | Ballman and Drummond 2017 | |
|  |  | *Prunus virginiana* | NS Canada | Little et al. 2017 | |
|  |  |  | MI USA | Leach et al. 2019 | |
|  |  |  | BC Canada | Thistlewood et al. 2019 | |
|  |  | *Prunus yedoensis* | Japan | Kanzawa 1935 | |
|  |  |  | Japan | Kanzawa 1939 | |
|  |  |  | Japan | Sasaki and Sato 1995 | |
|  | Pyracantha | *Pyracantha spp.* | Netherlands | Kenis et al. 2016 | |
|  | Pyrus | *Pyrus calleryana* | France | Poyet et al. 2015 | |
|  |  | *Pyrus communis* | NS Canada | Little et al. 2019 | |
|  |  | *Pyrus sinensis* | Japan | Kanzawa 1939 | |
|  |  | *Pyrus* spp. | MI USA | Bal et al. 2017 | |
|  | Rosa | *Rosa acicularis* | Netherlands | Kenis et al. 2016 | |
|  |  | *Rosa canina* | Spain | Arnó et al. 2016 | |
|  |  |  | Netherlands | Kenis et al. 2016 | |
|  |  | *Rosa glauca* | Netherlands | Kenis et al. 2016 | |
|  |  | *Rosa pimpinellifolia* | Netherlands | Kenis et al. 2016 | |
|  |  | *Rosa rugose* | Netherlands | Kenis et al. 2016 | |
| **Family** | **Genus** | **Species** | **Location** | **Reference** | |
|  | Rubus | *Rubus allegheniensis* | MA USA | Elsensohn and Loeb 2018 | |
|  |  |  | NS Canada | Little et al. 2019 | |
|  |  | *Rubus armeniacus* | OR USA | Lee et al. 2015 | |
|  |  |  | MI USA | Leach et al. 2019 | |
|  |  | *Rubus caesius* | Italy | Kenis et al. 2016 | |
|  |  |  | Netherlands | Kenis et al. 2016 | |
|  |  |  | Italy | Tonina et al. 2016 | |
|  |  | *Rubus chamaemorus* | NS Canada | Little et al. 2020 | |
|  |  | *Rubus crataegifolius* | Japan | Mitsui et al. 2010 | |
|  |  | *Rubus fruticosus* | France | Poyet et al. 2015 | |
|  |  |  | Slovenia | Seljak et al. 2015 | |
|  |  |  | Italy | Kenis et al. 2016 | |
|  |  |  | Netherlands | Kenis et al. 2016 | |
|  |  |  | Switzerland | Kenis et al. 2016 | |
|  |  | *Rubus idaeus* | Argentina | Andreazza et al. 2017 | |
|  |  |  | Italy | Grassi et al. 2011 | |
|  |  |  | CA USA | Goodhue et al. 2012 | |
|  |  |  | France | Poyet et al. 2015 | |
|  |  |  | Slovenia | Seljak et al. 2015 | |
|  |  |  | Italy | Kenis et al. 2016 | |
|  |  |  | Netherlands | Kenis et al. 2016 | |
|  |  |  | Switzerland | Kenis et al. 2016 | |
|  |  |  | NS Canada | Little et al. 2017 | |
|  |  |  | Argentina | Andreazza et al. 2017 | |
|  |  |  | MN USA | Sward (Thesis) 2017 | |
|  |  |  | MI USA | Leach et al. 2019 | |
|  |  |  | NS Canada | Little et al. 2019 | |
|  |  |  | NS Canada | Little et al. 2020 | |
|  |  | *Rubus laciniata* | NS Canada | Little et al. 2017 | |
|  |  | *Rubus microphyllus* | Japan | Kanzawa 1939 | |
| **Family** | **Genus** | **Species** | **Location** | **Reference** | |
|  |  |  | Japan | Mitsui et al. 2010 | |
|  |  | *Rubus occidentalis* | MN USA | Sward (Thesis) 2017 | |
|  |  |  | MA USA | Elsensohn and Loeb 2018 | |
|  |  | *Rubus parvifolius (=triphyllus)* | Japan | Kanzawa 1939 | |
|  |  |  | Japan | Sasaki and Sato 1995 | |
|  |  | *Rubus phoenicolasius* | Switzerland | Kenis et al. 2016 | |
|  |  | *Rubus saxatilis* | Italy | Kenis et al. 2016 | |
|  |  |  | Italy | Tonina et al. 2016 | |
|  |  | *Rubus spectabilis* | OR USA | Lee et al. 2015 | |
|  |  | *Rubus strigosus* | MI USA | Leach et al. 2019 | |
|  |  | *Rubus* spp. | Italy | Grassi et al. 2011 | |
|  |  |  | Slovenia | Seljak et al. 2015 | |
|  |  |  | MI USA | Bal et al. 2017 | |
|  |  |  | ME USA | Ballman and Drummond 2017 | |
|  |  |  | AR USA | Herrera (Thesis) 2017 | |
|  |  |  | Sweden | Manduric 2017 | |
|  |  | *Rubus ulmifolius* | Spain | Arnó et al. 2016 | |
|  | Sorbus | *Sorbus aria* | Italy | Kenis et al. 2016 | |
|  |  | *Sorbus aucuparia* | Netherlands | Kenis et al. 2016 | |
|  |  | *Sorbus sitchensis* | OR USA | Lee et al. 2015 | |
|  | Crataegus | *Crataegus chrysocarpa* | Netherlands | Kenis et al. 2016 | |
|  |  | *Crataegus monogyna* | Netherlands | Kenis et al. 2016 | |
| Rubiaceae | Rubia | *Rubia peregrina* | France | Poyet et al. 2015 | |
| Rutaceae | Murraya | *Murraya paniculata* | FL USA | Plant Inspection Advisory 2010 | |
|  | Skimmia | *Skimmia japonica* | France | Poyet et al. 2015 | |
|  |  |  | Netherlands | Panel et al. 2018 | |
|  | Citrus | *Citrus sinensis* | Argentina | Lavagnino et al. 2018 | |
|  |  | *Citrus X sinensis* | CA USA | Haviland et al. 2016 | |
| Santalaceae | Viscum | *Viscum album* | France | Poyet et al. 2015 | |
|  |  |  | Germany | Briem et al. 2016 | |
| **Family** | **Genus** | **Species** | **Location** | **Reference** | |
|  |  |  | Netherlands | Panel et al. 2018 | |
| Solanaceae | Atropa | *Atropa belladonna* | France | Poyet et al. 2015 | |
|  | Lycium | *Lycium barbarum* | Italy | Kenis et al. 2016 | |
|  | Physalis | *Physalis alkekengi* | France | Poyet et al. 2015 | |
|  | Solanum | *Solanum carolinense* | MI USA | Leach et al. 2019 | |
|  |  | *Solanum chenopodioides* | Spain | Arnó et al. 2016 | |
|  |  | *Solanum dulcamara* | MI USA | Lee et al. 2015 | |
|  |  |  | OR USA | Lee et al. 2015 | |
|  |  |  | France | Poyet et al. 2015 | |
|  |  |  | Spain | Arnó et al. 2016 | |
|  |  |  | Netherlands | Kenis et al. 2016 | |
|  |  |  | Switzerland | Kenis et al. 2016 | |
|  |  |  | ME USA | Ballman and Drummond 2017 | |
|  |  |  | MA USA | Elsensohn and Loeb 2018 | |
|  |  |  | MI USA | Leach et al. 2019 | |
|  |  | *Solanum luteum* | Spain | Arnó et al. 2012 | |
|  |  | *Solanum lycopersicum* | Japan | Kanzawa 1935 | |
|  |  |  | FL USA | Plant Inspection Advisory 2010 | |
|  |  |  | OR USA | Lee et al. 2015 | |
|  |  | *Solanum nigrum* | Spain | Arnó et al. 2016 | |
|  |  |  | Netherlands | Kenis et al. 2016 | |
|  |  |  | Switzerland | Kenis et al. 2016 | |
|  |  |  | France | Poyet et al. 2015 | |
|  |  | *Solanum tuberosum* | France | Poyet et al. 2015 | |
|  |  | *Solanum villosum* | Spain | Arnó et al. 2012 | |
| Taxaceae | Taxus | *Taxus baccata* | Italy | Kenis et al. 2016 | |
|  |  |  | Netherlands | Kenis et al. 2016 | |
|  |  |  | Switzerland | Kenis et al. 2016 | |
|  |  |  | France | Poyet et al. 2015 | |
|  | Torreya | *Torreya nucifera* | Japan | Mitsui et al. 2010 | |
| **Family** | **Genus** | **Species** | **Location** | **Reference** | |
| Thymelaeaceae | Daphne | *Daphne mezereum* | Italy | Kenis et al. 2016 | |
|  |  |  | Italy | Tonina et al. 2016 | |
| Vitaceae | Ampelopsis | *Ampelopsis glandulosa* brevipedunculata | AR USA | Herrera (Thesis) 2017 | |
|  | Parthenocissus | *Parthenocissus quinquefolia* | Switzerland | Kenis et al. 2016 | |
|  |  |  | MI USA | Leach et al. 2019 | |
|  | Vitis | *Vitis labrusca* | Slovenia | Seljak et al. 2015 | |
|  |  | *Vitis rotundifolia* | GA USA | Grant and Sial 2016 | |
|  |  | *Vitis* spp. | MI USA | Bal et al. 2017 | |
|  |  |  | Sweden | Manduric 2017 | |
|  |  |  | MI USA | Leach et al. 2019 | |
|  |  | *Vitis vinifera* | Italy | Grassi et al. 2011 | |
|  |  |  | Slovenia | Seljak et al. 2015 | |
|  |  |  | Italy | Kenis et al. 2016 | |
|  |  |  | NS Canada | Little et al. 2019 | |

**Supplementary Table S2**. Taxonomic classification of *D. suzukii* host plants using APG IV system (**The Angiosperm Phylogeny Group 2016**).

| **Division** | **Class** | **Subclass** | **Superclade** | **Clade** | **Subclade** | **Order** | **Family** | **Genus** | **# Species** |
| --- | --- | --- | --- | --- | --- | --- | --- | --- | --- |
| Gymnospermae | Pinopsida |  |  |  |  | Pinales | Taxaceae | Taxus | 1 |
| (Pinophyta) |  |  |  |  |  |  |  | Torreya | 1 |
|  |  |  |  |  |  |  |  |  |  |
| Angiospermae | Mesangiospermae | Magnoliids |  |  |  | Laurales | Lauraceae | Lindera | 1 |
|  |  | Monocots | Alismatids |  |  | Alismatales | Araceae | Arum | 2 |
|  |  |  | Liliods |  |  | Dioscoreales | Dioscoreaceae | Dioscorea | 1 |
|  |  |  |  |  |  | Liliales | Melanthiaceae | Paris | 1 |
|  |  |  |  |  |  | Asparagales | Asparagaceae | Polygonatum | 1 |
|  |  |  |  |  |  |  | Iridaceae | Iris | 1 |
|  |  |  | Commelinids |  |  | Arecales | Arecaceae | Butia | 1 |
|  |  | Eudicots |  |  |  | Buxales | Buxaceae | Sarcococca | 1 |
|  |  |  |  |  |  | Ranunculales | Berberidaceae | Berberis | 3 |
|  |  |  |  |  |  |  | Menispermaceae | Cocculus | 1 |
|  |  |  | Superrosids |  |  | Saxifragales | Grossulariaceae | Ribes | 6 |
|  |  |  |  | Rosids |  | Vitales | Vitaceae | Ampelopsis | 1 |
|  |  |  |  |  |  |  |  | Parthenocissus | 1 |
|  |  |  |  |  |  |  |  | Vitis | 3 |
|  |  |  |  |  | Fabids | Cucurbitales | Cucurbitaceae | Bryonia | 1 |
|  |  |  |  |  |  |  |  | Cucurbita | 1 |
|  |  |  |  |  |  | Fagales | Myricaceae | Myrica | 1 |
|  |  |  |  |  |  | Rosales | Rosaceae | Amelanchier | 3 |
|  |  |  |  |  |  |  |  | Aronia | 1 |
|  |  |  |  |  |  |  |  | Cotoneaster | 6 |
|  |  |  |  |  |  |  |  | Duchesnea | 1 |
|  |  |  |  |  |  |  |  | Eriobotrya | 1 |
|  |  |  |  |  |  |  |  | Fragaria x ananassa hybrid | 1 |
|  |  |  |  |  |  |  |  | Fragaria | 1 |
|  |  |  |  |  |  |  |  | Malus | 4 |
|  |  |  |  |  |  |  |  | Photinia | 3 |
|  |  |  |  |  |  |  |  | Prunus | 21 |
|  |  |  |  |  |  |  |  | Pyracantha | 1 |
|  |  |  |  |  |  |  |  | Pyrus | 3 |
|  |  |  |  |  |  |  |  | Rosa | 5 |
|  |  |  |  |  |  |  |  | Rubus | 16 |
|  |  |  |  |  |  |  |  | Sorbus | 3 |
|  |  |  |  |  |  |  |  | Crataegus | 2 |
|  |  |  |  |  |  |  | Elaeagnaceae | Elaeagnus | 3 |
|  |  |  |  |  |  |  |  | Hippophae | 1 |
|  |  |  |  |  |  |  | Rhamnaceae | Frangula | 3 |
|  |  |  |  |  |  |  |  | Rhamnus | 3 |
|  |  |  |  |  |  |  | Moraceae | Ficus | 1 |
|  |  |  |  |  |  |  |  | Morus | 5 |
|  |  |  |  |  | Malvids | Malvales | Thymelaeaceae | Daphne | 1 |
|  |  |  |  |  |  | Myrtales | Myrtaceae | Acca | 1 |
|  |  |  |  |  |  |  |  | Psidium | 2 |
|  |  |  |  |  |  |  |  | Punica | 1 |
|  |  |  |  |  |  |  |  | Eugenia | 2 |
|  |  |  |  |  |  |  | Onagraceae | Fuchsia | 1 |
|  |  |  |  |  |  | Sapindales | Rutaceae | Murraya | 1 |
|  |  |  |  |  |  |  |  | Skimmia | 1 |
|  |  |  |  |  |  |  |  | Citrus | 2 |
|  |  |  | Superastrids |  |  | Caryophyllales | Phytolaccaceae | Phytolacca | 2 |
|  |  |  |  |  |  |  | Basellaceae | Basella | 1 |
|  |  |  |  |  |  | Santalales | Santalaceae | Viscum | 1 |
|  |  |  |  | Asterids |  | Cornales | Cornaceae | Alangium | 1 |
|  |  |  |  |  |  |  |  | Cornus | 10 |
|  |  |  |  |  |  | Ericales | Ebenaceae | Diospyros | 1 |
|  |  |  |  |  |  |  | Actinidiaceae | Actinidia | 2 |
|  |  |  |  |  |  |  | Ericaceae | Arbutus | 1 |
|  |  |  |  |  |  |  |  | Empetrum | 1 |
|  |  |  |  |  |  |  |  | Vaccinium | 12 |
|  |  |  |  |  |  |  |  | Gaultheria | 3 |
|  |  |  |  |  | Lamiids | Gentianales | Rubiaceae | Rubia | 1 |
|  |  |  |  |  |  | Garryales | Garryaceae | Aucuba | 1 |
|  |  |  |  |  |  | Solanales | Solanaceae | Atropa | 1 |
|  |  |  |  |  |  |  |  | Lycium | 1 |
|  |  |  |  |  |  |  |  | Physalis | 1 |
|  |  |  |  |  |  |  |  | Solanum | 8 |
|  |  |  |  |  |  | Lamiales | Lamiaceae | Callicarpa | 1 |
|  |  |  |  |  |  |  | Oleaceae | Ligustrum | 2 |
|  |  |  |  |  | Campanulids | Apiales | Araliaceae | Hedera | 1 |
|  |  |  |  |  |  | Aquifoliales | Aquifoliaceae | Ilex | 1 |
|  |  |  |  |  |  | Dipsacales | Adoxaceae | Sambucus | 4 |
|  |  |  |  |  |  |  |  | Viburnum | 5 |
|  |  |  |  |  |  |  | Caprifoliaceae | Lonicera | 12 |
|  |  |  |  |  |  |  |  | Symphoricarpos | 1 |

**References**

**Andreazza, F., D. Bernardi, M. Botton, and D. E. Nava. 2015.** Índice de infestação de *Drosophila suzukii* e *Zaprionus indianus* (diptera: drosophilidae) em frutíferas nativas no município de Pelotas., p. 4p. *In* Embrapa Uva e Vinho-Artigo em anais de congresso (ALICE). In: Congresso de Iniciação Científica, 24., e Encontro da Pós-Graduação, 17.

**Andreazza, F., D. Bernardi, R. S. S. dos Santos, F. R. M. Garcia, E. E. Oliveira, M. Botton, and D. E. Nava. 2017.** *Drosophila suzukii* in Southern neotropical region: Current status and future perspectives. Neotropical Entomol. 46: 591–605.

**Angiosperm Phylogeny Group. 2016.** An update of the Angiosperm Phylogeny Group classification for the orders and families of flowering plants: APG IV. Botanical Journal of the Linnean Society. 181.

**Arnó, J., J. Riudavets, and R. Gabarra. 2012.** Survey of host plants and natural enemies of *Drosophila suzukii* in an area of strawberry production in Catalonia (northeast Spain). IOBC/WPRS Bulletin. 80: 29–34.

**Arnó, J., M. Solà, J. Riudavets, and R. Gabarra. 2016.** Population dynamics, non-crop hosts, and fruit susceptibility of *Drosophila suzukii* in Northeast Spain. J Pest Sci. 89: 713–723.

**Bal, H. K., C. Adams, and M. Grieshop. 2017.** Evaluation of off-season potential breeding sources for Spotted wing Drosophila (*Drosophila suzukii* Matsumura) in Michigan. J Econ Entomol. 110: 2466–2470.

**Ballman, E. S., and F. A. Drummond. 2017.** Infestation of wild fruit by *Drosophila suzukii* surrounding Maine wild blueberry fields. J Agric Urban Entomol. 33: 61–70.

**Briem, F., A. Dominic, B. Golla, C. Hoffmann, C. Englert, A. Herz, and H. Vogt. 2018.** Explorative data analysis of *Drosophila suzukii* trap catches from a seven-year monitoring program in Southwest Germany. Insects. 9: 125.

**Diepenbrock, L. M., and D. R. McPhie. 2018.** Use of a novel host plant family, Basallaceae, by *Drosophila suzukii* (Diptera: Drosophilidae). Proc Entomol Soc Wash. 120: 255–259.

**Elsensohn, J., and G. Loeb. 2018.** Non-crop host sampling yields insights into small-scale population dynamics of *Drosophila suzukii* (Matsumura). Insects. 9: 5.

**González, G., A. L. Mary, and B. Goñi. 2015.** *Drosophila suzukii* (Matsumura) found in Uruguay. Drosoph Inf Serv. 98: 103–107.

**Goodhue, R. E., M. Bolda, D. Farnsworth, J. C. Williams, and F. G. Zalom. 2011.** Spotted wing drosophila infestation of California strawberries and raspberries: economic analysis of potential revenue losses and control costs. Pest Management Science. 67: 1396–1402.

**Grant, J. A., and A. A. Sial. 2016.** Potential of Muscadine grapes as a viable host of *Drosophila suzukii* (Diptera: Drosophilidae) in blueberry-producing regions of the Southeastern United States. J Econ Entomol. 109: 1261–1266.

**Grassi, A., L. Giongo, and L. Palmieri. 2011.** Drosophila (Sophophora) suzukii (Matsumura), new pest of soft fruits in Trentino (North-Italy) and in Europe. IOBC/wprs Bull. 70: 121–128.

**Grassi, A., A. Gottardello, D. T. Dalton, G. Tait, D. Rendon, C. Ioriatti, D. Gibeaut, M. V. Rossi Stacconi, and V. M. Walton. 2018.** Seasonal reproductive biology of *Drosophila suzukii* (Diptera: Drosophilidae) in temperate climates. Environ Entomol. 47: 166–174.

**Haviland, D. R., J. L. Caprile, S. M. Rill, K. A. Hamby, and J. A. Grant. 2016.** Phenology of spotted wing drosophila in the San Joaquin Valley varies by season, crop and nearby vegetation. Calif Agric. 70: 24–31.

**Herrera, L. 2017.** Seasonality and management of Spotted wing Drosophila on berry crops and wild hosts in Arkansas. *Theses and Dissertations.* 2610. Retrieved from ScholarWorks@UARK , https://scholarworks.uark.edu/etd/2610

**Hietala-Henschell, K., E. Pelton, and C. Guédot. 2017.** Susceptibility of Aronia ( *Aronia melanocarpa* ) to *Drosophila suzukii* (Diptera: Drosophilidae). J Kans Entomol Soc. 90: 162–170.

**Kanzawa, T. 1935.** Research into the fruit-fly *Drosophila suzukii* Matsumura (preliminary report). Yamanashi Prefecture Agricultural Experiment Station Report.

**Kanzawa, T. 1939.** Studies on *Drosophila suzukii* Mats. Kofu, Yamanashi Agricultural Experiment Station. Rev Appl Entomol. 29: 622.

**Kenis, M., L. Tonina, R. Eschen, B. van der Sluis, M. Sancassani, N. Mori, T. Haye, and H. Helsen. 2016.** Non-crop plants used as hosts by *Drosophila suzukii* in Europe. J Pest Sci. 89: 735–748.

**Kinjo, H., Y. Kunimi, T. Ban, and M. Nakai. 2013.** Oviposition efficacy of <I>Drosophila suzukii</I> (Diptera: Drosophilidae) on different cultivars of blueberry. J Econ Entomol. 106: 1767–1771.

**Knipp, R. 2018.** Spotted wing Drosophila (*Drosophila suzukii*) in Arkansas: Winter morphs, wild hosts, and fungal control. *Theses and Dissertations*. 2966. Retrieved from ScholarWorks@UARK ,https://scholarworks.uark.edu/etd/2966

**Lasa, R., E. Tadeo, L. A. Dinorín, I. Lima, and T. Williams. 2017.** Fruit firmness, superficial damage, and location modulate infestation by *Drosophila suzukii* and *Zaprionus indianus* : the case of guava in Veracruz, Mexico. Entomol Exp Appl. 162: 4–12.

**Lavagnino, N. J., B. M. Díaz, L. I. Cichón, G. J. De la Vega, S. A. Garrido, J. D. Lago, and J. J. Fanara. 2018.** New records of the invasive pest *Drosophila suzukii* (Matsumura) (Diptera: Drosophilidae) in the South American continent. Rev Soc Entomol Argent. 77: 27–31.

**Leach, H., J. R. Hagler, S. A. Machtley, and R. Isaacs. 2019.** Spotted wing drosophila ( *Drosophila suzukii* ) utilization and dispersal from the wild host Asian bush honeysuckle ( *Lonicera* spp.). Agric For Entomol. 21: 149–158.

**Lee, J. C., A. J. Dreves, A. M. Cave, S. Kawai, R. Isaacs, J. C. Miller, S. Van Timmeren, and D. J. Bruck. 2015.** Infestation of wild and ornamental noncrop fruits by *Drosophila suzukii* (Diptera: Drosophilidae). Ann Entomol Soc Am. 108: 117–129.

**Little, C. M., T. W. Chapman, and N. K. Hillier. 2018.** Effect of color and contrast of highbush blueberries to host-finding behavior by *Drosophila suzukii* (Diptera: Drosophilidae). Environ Entomol. 47: 1242–1251.

**Little, C. M., T. W. Chapman, D. L. Moreau, and N. K. Hillier. 2017.** Susceptibility of selected boreal fruits and berries to the invasive pest *Drosophila suzukii* (Diptera: Drosophilidae). Pest Manag Sci. 73: 160–166.

**Little, C. M., P. L. Dixon, T. W. Chapman, and N. K. Hillier. 2020.** Role of fruit characters and colour on host selection of boreal fruits and berries by *Drosophila suzukii* (Diptera: Drosophilidae). The Canadian Entomologist. 1–17.

**Little, C. M., E. Rand, M. MacIsaac, L. Charbonneau, and N. K. Hillier. 2019.** FlySpotter: using citizen science to identify range expansion and fruit at risk from *Drosophila suzukii* in Nova Scotia and Newfoundland and Labrador. J Acadian Entomol Soc. 15: 27–39.

**Manduric, S. 2017.** *Drosophila suzukii* - experiences from the fly’s northernmost inhabited region (from the first record to two years after the detection). IOBC/WPRS Bulletin. 123: 150–156.

**Mitsui, H., K. Beppu, and M. T. Kimura. 2010.** Seasonal life cycles and resource uses of flower- and fruit-feeding drosophilid flies (Diptera: Drosophilidae) in central Japan. Entomol Sci. 13: 60–67.

**Mitsui, H., K. H. Takahashi, and M. T. Kimura. 2006.** Spatial distributions and clutch sizes of Drosophila species ovipositing on cherry fruits of different stages. Popul Ecol. 48: 233–237.

**Panel, A., L. Zeeman, B. van der Sluis, P. van Elk, B. Pannebakker, B. Wertheim, and H. Helsen. 2018.** Overwintered *Drosophila suzukii* are the main source for infestations of the first fruit crops of the season. Insects. 9: 145.

**Plant Inspection Advisory. 2010.** Update for Spotted wing Drosophila, *Drosophila suzukii* and potential on blueberries. Memo to: Bureau of Plant & Apiary Inspectors and Supervisors, Florida.

**Poyet, M., P. Eslin, M. Héraude, V. Le Roux, G. Prévost, P. Gibert, and O. Chabrerie. 2014.** Invasive host for invasive pest: when the Asiatic cherry fly ( *Drosophila suzukii* ) meets the American black cherry ( *Prunus serotina* ) in Europe: When *D.* *suzukii* meets *P.* *serotina*. Agric For Entomol. 16: 251–259.

**Poyet, M., V. Le Roux, P. Gibert, A. Meirland, G. Prévost, P. Eslin, and O. Chabrerie. 2015.** The wide potential trophic niche of the Asiatic fruit fly *Drosophila suzukii*: The key of its invasion success in temperate Europe? PLOS ONE. 10: e0142785.

**Sasaki, M. (Fukushima-ken F. T. E. S. (Japan)), and R. Sato. 1995.** Bionomics of the cherry drosophila, *Drosophila suzukii* Matsumura (Diptera: Drosophilidae) in Fukushima Prefecture [Japan], 3: Life cycle. Annu Rep Soc Plant Protect N Japan.

**Seljak, G., M. Jančar, and M. Rot. 2015.** Razširjenost plodove vinske mušice (Drosophila suzukii) v Sloveniji in njena populacijska dinamika v Obdobju 2011-2014. Zbornik predavanj in referatov. 12: 43–48.

**Souza, G. K., T. G. Pikart, V. L. de Oliveira, P. Boff, and M. I. C. Boff. 2017.** *Acca sellowiana* (Myrtaceae): A new alternative host for *Drosophila suzukii* (Diptera: Drosophilidae) in Brazil. Fla Entomol. 100: 190–191.

**Steffan, S. A., J. C. Lee, M. E. Singleton, A. Vilaire, D. B. Walsh, L. S. Lavine, and K. Patten. 2013.** Susceptibility of cranberries to *Drosophila suzukii* (Diptera: Drosophilidae). J Econ Entomol. 106: 2424–2427.

**Stewart, T. J., X.-G. Wang, A. Molinar, and K. M. Daane. 2014.** Factors limiting peach as a potential host for *Drosophila suzukii* (Diptera: Drosophilidae). J Econ Entomol. 107: 1771–1779.

**Sward, G. 2017.** Evaluating host plant use by Spotted wing Drosophila, *Drosophila suzukii*, in Minnesota. *Theses and Dissertations*. Retrieved from the University of Minnesota Digital Conservancy,  http://hdl.handle.net/11299/188757

**The Angiosperm Phylogeny Group, M. W. Chase, M. J. M. Christenhusz, M. F. Fay, J. W. Byng, W. S. Judd, D. E. Soltis, D. J. Mabberley, A. N. Sennikov, P. S. Soltis, and P. F. Stevens. 2016.** An update of the Angiosperm Phylogeny Group classification for the orders and families of flowering plants: APG IV. Botanical Journal of the Linnean Society. 181: 1–20.

**Thistlewood, H. M. A., B. Rozema, and S. Acheampong. 2019.** Infestation and timing of use of non-crop plants by *Drosophila suzukii* (Matsumura) (Diptera: Drosophilidae) in the Okanagan Basin, Canada. Can Entomol. 151: 34–48.

**Tonina, L., N. Mori, F. Giomi, and A. Battisti. 2016.** Development of *Drosophila suzukii* at low temperatures in mountain areas. J Pest Sci. 89: 667–678.

**Turcotte, R. M., C. Larcenaire, R. Long, D. K. H. Martin, and L. Barringer. 2018.** The Spotted wing Drosophila, *Drosophila suzukii* (Diptera: Drosophilidae): A new pest of concern for black cherry, *Prunus serotina,* on the High Allegheny Plateau in Pennsylvania. Entomol News. 127: 390–399.

**Yu, D., F. G. Zalom, and K. A. Hamby. 2013.** Host status and fruit odor response of *Drosophila suzukii* (Diptera: Drosophilidae) to figs and mulberries. J Econ Entomol. 106: 1932–1937.

**Yukinari, M. 1988.** Drosophilid flies injurious to the fruits of wax-myrtle, *Myrica rubra* Sied et Zucc [in Japan]. Japanese J Appl Entomol Zool.
